# Parents’ experiences of an e-health intervention implemented in pediatric healthcare: a qualitative study

**DOI:** 10.1186/s12913-019-4643-7

**Published:** 2019-11-05

**Authors:** Ingrid Larsson, Petra Svedberg, Susann Arvidsson, Jens M. Nygren, Ing-Marie Carlsson

**Affiliations:** School of Health and Welfare, Halmstad University, PO Box 823, S-30118 Halmstad, Sweden

**Keywords:** Children, E-health intervention, Grounded theory, Parents, Participation

## Abstract

**Background:**

The growing field of participation in healthcare has the potential to provide a number of benefits for children, patients, healthcare professionals and also the healthcare systems. According to the Convention on the Rights of the Child (UNCRC), children have the right to participate in their own healthcare and make their voice heard. Children’s opportunities for understanding their conditions, sharing their views and participating in decisions regarding their care depend on healthcare professionals but also on parents’ ability to communicate and include children. E-health solutions can remove barriers to children’s communication with healthcare professionals. The aim of this study was to explore parents’ perspectives on the outcomes of an e-health solution, Sisom, used by children during healthcare appointments.

**Methods:**

The empirical data is based on interviews with 16 parents. In the present study constructivist, grounded theory was chosen as the method.

**Results:**

The theory of enhancing participation, by orientating communication about healthcare towards the voice of the child instead of the parents, summarizes the process of how the outcome of Sisom for children lead to enhanced participation, by making the child the main actor and an agent in his/her own healthcare. The facilitators for achieving participation in Sisom were four interrelated outcomes; engaging, voice-guarding, raising awareness and integrity preserving. In addition to generating increased participation, it emerged that the use of Sisom also initiated a process, which was evident in all four subcategories that facilitated the child in coping with the experience of having an illness.

**Conclusions:**

We conclude, that Sisom orientated communication about healthcare towards the voice of the child instead of the parents as well as including the child in the dialogue with the healthcare professional and thus increasing the child’s participation and human rights.

## Background

The growing field of participation in healthcare has the potential to provide a number of benefits for children, patients, healthcare professionals and also healthcare systems. Despite a general acceptance regarding the importance of participation, challenges remain in translating such ambitions into practice, especially when it comes to children. The right of children to be listened to and to be involved in all situations that affect them is regulated by the Convention on the Rights of the Child (UNCRC) [1] and this is of particular importance when the child is in a vulnerable situation and in need of healthcare. Despite this, healthcare professionals often fail to provide opportunities for children to understand their conditions, share their views and participate in decisions regarding their care [2, 3]. Similarly, it is common that the parent role marginalizes and excludes children in healthcare [4, 5] even if parents play an important role in decision-making and preparing children for healthcare encounters [6]. Optimal communication between children and healthcare professionals is crucial for children’s participation in their own healthcare [7], allowing them to be listened to and being given explanations in a way they understand [8] as well as being an actor in decision-making [3, 9]. Other factors that impact this communication are parents’ values, personal wishes and sociocultural background, healthcare professionals’ attitudes, the capacity of the child, and the seriousness of the matter being discussed [9, 10].

The use of e-health solutions is expected to improve communication for children in healthcare situations [11] and can complement traditional patient education and support for children and their parents [12]. Novel ways of providing e-health solutions offer opportunities for children to communicate more in a person-centered manner and in private with healthcare professionals and are, according to parents, removing barriers for children’s communication with healthcare professionals [13]. Moreover, children find it is easier and more fun to respond to assessment items provided through mobile devices than orally or on paper [14]. Mobile solutions increase motivation and possibility for children of different ages and with different functional variations to participate [15]. However, difficulties and challenges have been widely reported when efforts have been made to implement and evaluate e-health solutions in practice [16–18].

Sisom (Norwegian acronym “Si det som det er” or “Tell it how it is”) is an interactive digital assessment and communication tool for children (6–12 years) with a child-friendly interface [14, 19]. Sisom helps children describe their life situation during a discovery journey among islands to promote communication with healthcare professionals [20]. This enables healthcare professionals to better understand and respond to the child with appropriate healthcare [19, 21]. However, the evaluation of its implementation in clinical practice and its effects on healthcare and outcomes is still lacking. The current qualitative study is part of an implementation project where Sisom has been implemented at four pediatric care centers in Sweden [22]. The aim of this study was to explore parents’ perspectives on the outcomes of an e-health solution, Sisom, used by children during healthcare appointments.

## Method

### Design

A constructivist, grounded theory was chosen for the present study since this method is well-suited for exploring processes. The method allows the researcher to examine the participants’world of meaning and action from the participants´ perspectives in a specific context [23]. Constructivist grounded theory is a constant comparative method that uses an inductive approach with iterative logic of abduction to check and refine the development of categories [23].

### Setting and intervention

An implementation study [22] was conducted at four pediatric care centers at three different hospitals in Sweden in 2016–2017. The purpose of the implemented e-health intervention, Sisom, was to increase children’s participation in their own healthcare by helping them identify and voice their concerns. During the children’s appointments at the pediatric units, they used Sisom on a tablet where they made a virtual journey to five islands; at the hospital, about managing things, my body, thoughts and feelings, and things one can be afraid of. Each island contained questions (84 in total) representing different dimensions of the child’s life situation. For each question, the child selected the level of agreement using five differently colored smileys complemented with the text; “No problem,” “A little,” “Some,” “A lot,” and “Don’t know”. A child-friendly report was printed out with the answers to the questions and formed the basis for the clinical dialogue between each child and his/her healthcare professional as part of the child’s healthcare process. The intervention lasted for 6 months, during which each child used Sisom on at least two occasions. A total of 46 children were included and 33 children completed the intervention.

### Participants

The participants in this study were parents to children who have completed the intervention at the pediatric care centers. A purposeful sample of 17 parents was contacted, one declined and 16 agreed to participate (Table 1).

**Table 1:** The characteristics of the parents and their children

Participating parents (n)	16
Sex	
Female	13
Male	3
Age (years)	
Median	41
Range	32–51
Civil status	
Married/cohabit	13
Single	3
Educational level	
Primary	
Secondary level	9
College/University	7
Employment	
Working full or part-time	13
Sick-leave/unemployed/parental leave	3
Experience of pediatric care (years)	
Median	7
Range	1–12
Pediatric care centers	
Pediatric counselor out and inpatient care (Centre A)	2
Pediatric counselor out and inpatient care (Centre B)	6
Pediatric oncology outpatient care (Centre C)	4
Pediatric neurology outpatient care (Centre D)	4
Born in Sweden	16
Children of the participants (n)	16
Sex	
Female	8
Male	8
Age (years)	
Median	10
Range	6–13
Diseases	
Cancer	7
Diabetes	1
Heart diseases	1
Hematologic diseases	1
Neurological diseases	5
Other	1

### Data collection

Individual interviews with parents, which either took place at the university or at the local hospitals or in the parent’s homes, were carried out between January 2017 and November 2017. The first author (IL) conducted the first 15 interviews and the third author (SA) conducted the last interview. Due to long distances, some of the interviews were performed by video conferencing tools (Skype or Face-Time). The parents were encouraged to speak openly about their experiences related to the child’s use of Sisom during appointments at the hospital. Each interview was based on ongoing analysis and was guided by questions pertaining to the emerging theory. The interviews were digitally recorded, lasted between 45 and 90 min and were transcribed verbatim.

### Data analysis

In accordance with grounded theory analysis, data collection and data analysis with coding of data were carried out in conjunction with analysis [24] There are three types of coding in a constructivist grounded theory study: 1) initial coding, 2) focused coding and 3) theoretical coding [23]. During the whole analysis, questions were asked of the data and constant comparison of data was used to refine the data, relationships, and inter-relationships. Furthermore, writing memos written during the whole analysis process. Memos act as researchers´ latent thoughts and are vital to grounded theory.

The analysis in the present study started with initial coding when the researcher worked with the data directly. Interview transcripts were read line-by-line separately by two of the authors (IL and I-MC) [23]. Incidents with relevance to the aim were fractured, condensed and coded into in vivo codes. The codes were written as gerunds and maintained close to data and if possible, using the parent’s own words. Initial coding guided the following interviews, for example, the initial code “taking a step back” guided subsequent interviews, and thus, the authors started asking questions about the parent’s own feelings when they had noticed that questions were put to their child instead of them. The second stage of the analysis was the focused coding where the coded incidents were compared and pieced together based on similarities. The initial codes “not taking anything for granted” and “some things are hard to talk about” formed a pattern together with further codes. Together these constructed a concept, “shedding light on the child’s thoughts” that finally ended up as the subcategory raising awareness. The final analysis, the theoretical coding began when the core category emerged i.e. that Sisom orientated communication about healthcare towards the voice of the child. During the continuing analysis process the constructed set of concepts was saturated in terms of properties, characteristics, and dimensions through constant comparison. When no new properties or dimensions emerged from continued coding and comparison, theoretical saturation was met, which meant that the concepts have achieved theoretical saturation. At this point, the theoretical sorting of memos was performed and integrated into the theory which moved the analysis from the level of descriptive to that of explanation.

## Results

### The theory of enhancing participation by orientating communication about healthcare towards the voice of the child instead of the parents

**Fig. 1:**
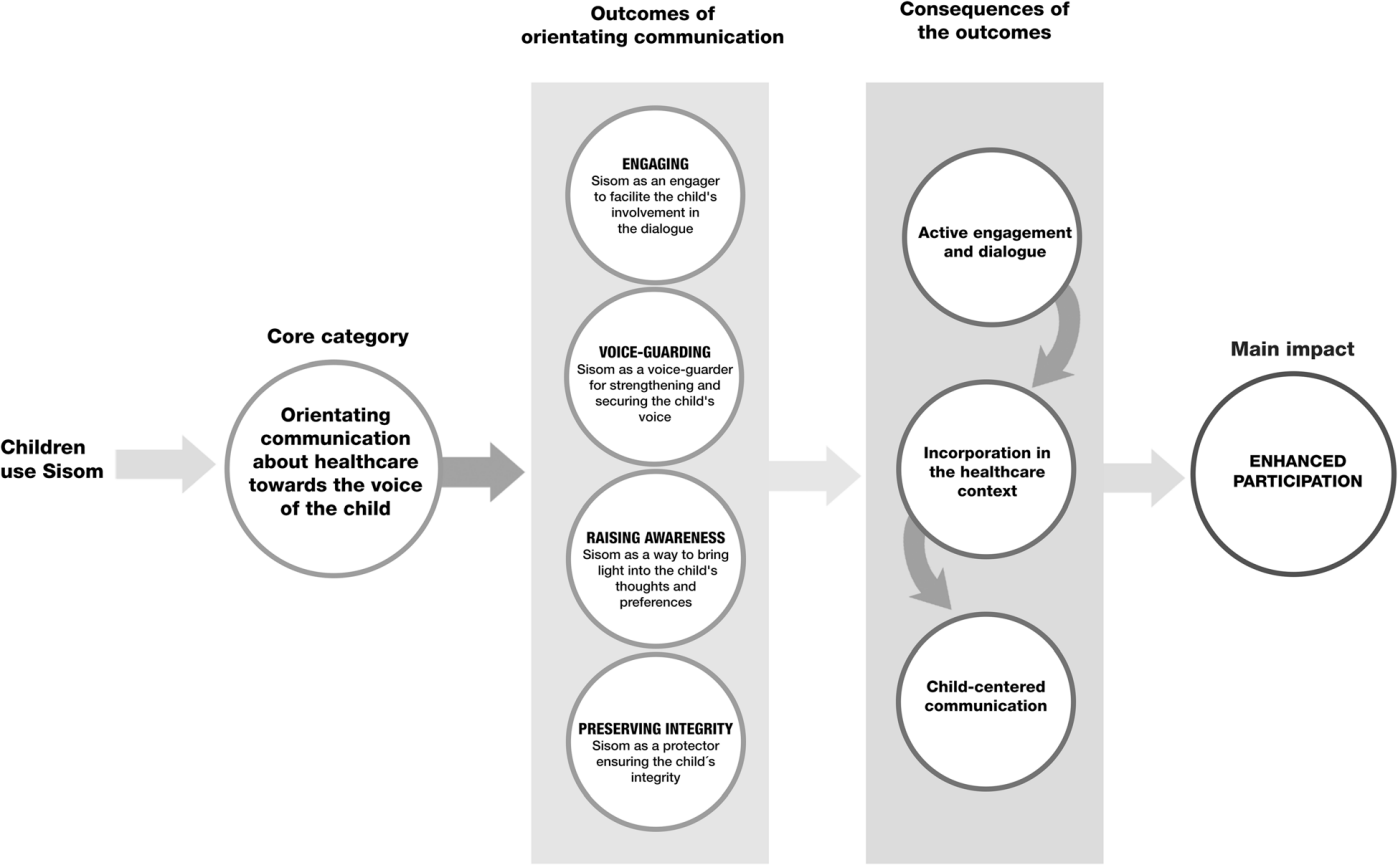
The theory of enhancing participation by orientating communication about healthcare towards the voice of the child instead of the parents

The theory represents how Sisom, used during pediatric healthcare appointments, increased children’s participation in their healthcare, by focusing communication with the child and thus changing the direction of communication away from the parents and towards their child. The core category, *orientating communication about healthcare towards the voice of the child*, resulted in four outcomes; engaging, voice-guarding, raising awareness, and preserving the integrity. As a consequence of these outcomes, an active engagement and dialogue were created that incorporated the child in the healthcare context and led to an achieved child-centered communication. All together this process led to enhanced participation (Fig. 1).

In addition to generating enhanced participation, it emerged that the use of Sisom also initiated the processing of experiences and emotions in relation to the child’s illness and care, which was evident in all four subcategories. The parents emphasized that enabling the child to reveal feelings and being able to talk about difficult topics, helped the child to cope with the illness - a prerequisite for the child to move on.

### The core category orientating communication about healthcare towards the voice of the child

The core category orientating communication about healthcare towards the voice of the child summarized the process of how the use of Sisom lead to enhanced participation, by making the child the main actor and an agent in her/his pediatric healthcare and thus reducing the role of parents as advocates for their child.

The healthcare professionals were generally in a position to direct the dialogue between the parents, the child and themselves, prior to the e-health intervention. The parents described that the child was in the room, playing around and was a passive participant in the triadic dialogue. Parents usually received attention from the healthcare professionals and sometimes the child was completely left out of the dialogue during the appointment.

*“…Otherwise, it’s always the parents who talk. It’s not often, my children don’t say very much anyhow”. (No.8)*

The direction of the healthcare professional’s communication changed from the parents towards the child by using Sisom. This was interpreted as the initiation of a process of orientating towards child-centered care. The child became the focus of the communication and thus developed into an agent working together with the healthcare professionals.

*“It was new for us and we wanted to speak, wanted to be in charge sort of. On some appointments, I’m the one carrying the bag because our child can say what she thinks and we think that’s really good, which means that she dares to talk and stand there and go first so it’s good actually. It feels good that she’s the one who gets to speak”. (No.2)*

When the healthcare professionals focused on the child to a greater extent an atmosphere of a consultation exclusively for the child was created. Thus, the child had full attention, was listened to and taken seriously. This was an important outcome for parents knowing that the healthcare professionals cared and valued the child’s perspective of the situation. Moreover, having a parental role and power of attorney over a family member was considered a big responsibility. However, by making the child an agent in healthcare the parents were able to step back; take a breather. As a result of the child’s increased involvement, the parents’ feelings of responsibility and parental control were thus reduced, which was expressed as emotional relief. Furthermore, an additional advantage of being able to step back also meant preventing competing for the healthcare professional’s attention and avoiding talking above the child’s head.

*“Then they’re more active in their care. The children participate more….. Then they get a little more self-confidence, think that they’re a part of it and learn a bit more perhaps and something new and feel more competent and get this self-confidence if they can express themselves .. then we can take a step back as parents. Then they can express themselves a little and we don’t have to always join in”. (No.5)*

### Outcomes of orientating communication

#### Engaging

Engaging was the first step in enhancing participation by orientating communication about healthcare to the voice of the child instead of the parents. Sisom engaged and captured the child’s interest without pressure from others and was referred to as a communication starter for involving the children in their healthcare. Sisom thus became an intermediary in the dialogue. This resulted in the parents not having to encourage the child to talk. The parents could take a step back and remain in the background.

*“It’s perhaps easier to have a tablet than talking with words. The child clicks on it or looks at the picture instead of talking. The child may be able to express a little more about what it thinks and feels about the matter. The child thinks it fun to do it (Sisom) and is more involved in this than she was before .. Then the child is more involved and wants to be a part of it and perhaps decide and perhaps talk a little more about her care” (No.5)*

The parents described that the children enjoyed Sisom, the user-friendly interface and that the questions were addressed directly to the child. Even those who had problems in expressing themselves, or had a quiet personality, answered the questions more extensively, which was in contrast to the usual conversations where the parents had to answer the questions. This was a relief for the parents as they did not have to entice the child to speak. Moreover, children with cognitive deficits managed to concentrate, and complete all the questions without being bored and also wanted more questions, which surprised the parents. They experienced that Sisom captured the children’s interest and that answering the questions was like being in a competition with themselves and making them feel proud when being able to answer all of them.

*“exciting, fun and easy so that even my child who has ADHD became interested and could complete it himself”.* *(No.13)*

Sisom enabled the children to express their feelings and preferences in an easy way. This was described by the parents as being important for the child in order to process experiences and emotions in relation to their illness, care, and situation. Consequently, the children developed confidence in healthcare professionals and became more self-confident when they felt they were listened to. Parents saw this as a key to developing independence.

*The child’s voice was heard and in a way that was quite easy for him, which I felt my child thought. There was no problem. Making it this fun to answer such important questions but while it being easy can probably help the child to feel that he then finds it easy to express himself. It (Sisom) helps the child to feel strong and proud and his self-esteem grows…. There were actually quite easy questions that gave us quite a lot of information”. (No.11)*

#### Voice-guarding

Voice-guarding entailed strengthening and protecting the voice of the child and thus the child’s position in the healthcare situation. Voice-guarding was another step in enhancing participation by orientating communication about care towards the voice of the child instead of parents. Sisom ensured that the dialogue was embodied and was focusing on the child’s own experiences. This was described as a relief for the parents when they did not have to interpret the child’s thoughts and feelings. The question areas in Sisom also created prerequisites for a structure in the subsequent dialogue with relevant follow-up questions that ensured that the child’s voice was listened to.

*“One probably misses a lot, it’s impossible to explain how another person feels. I have to trust what I see. I don’t know what it’s like when my child is with others if you understand what I mean. She’s the only one who can feel it”.* *(No.4)*

Furthermore, voice-guarding included trusting and respecting the child as an expert and a capable person, thus strengthening the child’s position. The parents noticed that the child took the lead in the dialogue and that the questions were interpreted by the one it concerned. The parents perceived that the children’s answers were honest and related to their experiences, and not answers adapted to what the children believed would be the right ones that would satisfy adults. Even if some of the experiences could be emotionally difficult to talk about, the parents described that Sisom helped the children to process their feelings. Revealing feelings could be difficult and some children felt both sad and tired afterward, but the parents emphasized the importance for the child of being able to process the questions and answers and that being given the possibility to answer questions provided them with the chance of obtaining confirmation of their experiences.

*“One can have different experiences of the same thing but in this Sisom when my child answered himself then it became his own. Then the child answers what he thinks and not what he expects or thinks that I (as a parent) thinks …The child is strengthened in daring to ask things or talk about things or in understanding that his opinions are important or that he is allowed to participate and influence some parts”. (No.7)*

#### Raising awareness

Sisom was a way to shed light on the child’s thoughts and preferences, which enabled them to gain a holistic view of the child’s experiences. Raising awareness was also a step in enhancing participation by orientating communication about care towards the voice of the child instead of the parents. A large amount of valuable information about the child was generated by some quite simple questions, which also enhanced the children’s impact on the outcome of their story. The parents meant that they became aware of information that otherwise would not have been revealed. For example, Sisom pooled the child’s different worlds, such as healthcare and school environment and included questions adults would not usually dare to ask children, e.g. if they are afraid of dying or if they think that their parents are worried about them.

“ *...to talk with a nine-year-old about death, about survival, who one is and what happens now and such things. It (Sisom) became a good way for my child, I think. Just being able to express oneself in a different way. Because I know that we cried quite a lot in the beginning and my child didn’t want us to cry and told us that everything is OK. So then my child can’t tell us her parents how she feels and so perhaps this (Sisom) helped her to express herself”.* *(No.2)*

Furthermore, the parents described that Sisom generated concrete and clear answers from the child with differently colored smileys. With the permission of the child, his/her answers could be explicit, readable, and even printed out, and used as a basis for a more profound dialogue both between the child and the healthcare professionals but also between the child and the parents. Patterns could be recognized by comparing previous reports and used to tailor future decisions to the children’s experiences and preferences. Repeated use of Sisom together with the dialogue with healthcare professionals was a way for the child to be aware of and process their emotions and feelings. The parents described this as a prerequisite for the child to move on.

The parents expressed that their child’s inner thoughts and feelings could sometimes surprise them. This created an awareness of not taking anything for granted even though they were the child’s parents. However, information gained from Sisom raised awareness among the child, the parents and the healthcare professionals to adopt new perspectives, and to act in new ways to work together to tailor the child’s healthcare. Furthermore, new information and the subsequent dialogue led to the development of greater trust between the child and the parents, which helped them to talk about feelings of anxiety and fear, thus influencing the children’s ability to process their situation.

*“That the child also gets to know himself by answering the questions. That he knows, OK; I answered red smiley on this and then he knows that I don’t like this. So that the child can express how he feels about situations and he also knows that we know …So this (Sisom) makes one aware of one’s own thoughts and feelings about the child and the child sees that we (his parents) know”. (No.11)*

#### Preserving integrity

Preserving integrity entailed the integrity of the child’s inner feelings, thoughts, views, and wishes were assured, which was a crucial step in enhancing participation in communication about care. By using Sisom, the child controlled and dictated the communication. The child could invite the healthcare professional and the parents into the dialogue on their own terms by choosing whether either one or both could attend the subsequent dialogue. This was a prerequisite for creating trust and respect for what the child chose to talk about. It meant that Sisom informed parents and healthcare professionals about what was important for the child and what the child wanted to avoid talking about. Since the children were given the power to approve which questions should be answered, they were able to control the dialogue. Furthermore, the parents noticed that their child sometimes chose a green smiley when they knew that their child had problems with the issue raised by that question. The parents interpreted this as a conscious choice by the child to avoid a dialogue that the selection of a red smiley otherwise would have caused.

*“The child can choose which parts he will share, with whom and how deeply he goes into it, there’s nothing that obtrudes on him I think. Again it’s the interactive game and the tablet, it’s natural for them”. (No.15)*

The information in the report was dealt with as information only in relation to the child’s healthcare and the child chose whether the parents should be involved. By using Sisom the child was thus able to keep secrets and be assured that their integrity was respected. Keeping secrets was not described as a barrier for parents, instead, it could be seen as something positive for the parents if the child was able to entrust their thoughts and feelings to someone outside the family, such as the healthcare professionals. The parents experienced this as an important part of the child’s processing of the situation. As parents, they did not need to know everything about their child and they suggested that the dialogue could be benefitted by the parents not being in the room. The child could then instead invite the parents towards the end of the dialogue if he/she wanted. There was a tendency prior to using Sisom that the children tried to keep their feelings to themselves by not showing them or by omitting relevant information when the parents were present in the conversation. By using Sisom, they were able to answer honestly without thinking of protecting their parents or maintaining a brave front.

*“Because our child wanted to be by herself with the pediatric counselor. She didn’t want us to be in the room. And then they decided that there were some matters that we could discuss together so we could join them at the end”. (No.14)*

## Discussion

In this study, the generated theory of enhancing participation by orientating communication about healthcare towards the voice of the child instead of the parents, which was grounded in interview data from parents, explains how the use of the e-health solution Sisom changed communication patterns in healthcare situations. This theory incorporates the core category *orientating communication about healthcare towards the voice of the child, which* describes the process of how the use of Sisom enabled the child to be the main character and an agent in pediatric healthcare in order to achieve participation. In addition to the core category, four related subcategories emphasize how the parents experienced that the process promoted participation in pediatric healthcare by engaging and capturing the child’s interest without pressure from others, voice-guarding and protecting the child’s voice, raising awareness with a holistic view of the child’s experiences and preferences, and preserving the child’s integrity. Overall, children became more independent of their parents when they were given the opportunity to express themselves in an easy and child-friendly way and thus developed as human beings. This also resulted in relief for the parents when their responsibility for being an advocate for their child decreased. Sisom was thus successfully implemented from the parents’ perspective, which contributed to the translation of the children’s right for participation in practice [1, 25]. By using Sisom, the children became active and involved in the dialogue about their life situation, their experiences, and preferences in healthcare. The communication about healthcare was orientated towards the voice of the child while preserving the child’s integrity and thus contributing to a child-centered communication. This is in accordance with the framework of child-centered care where the children are respected and involved in communication by making their voice heard about needs and preferences and participating in decision-making [26]. The use of e-health communication tools in healthcare has been proposed to affect the role and expectations of the child, the parents, and the healthcare professionals and thus facilitate child-centered care [27, 28].

The broad spectrum of interventions and approaches for facilitating participation in pediatric healthcare is, however, primarily aimed at parents [29, 30]. In order for children to achieve meaningful participation in decision-making, appropriate interventions aimed directly at children should be developed where an appropriate level of understanding and an opportunity to express their own thoughts and feelings are required [7, 31]. However, a common challenge in pediatric healthcare is that oral communication and written information are often developed by and aimed for adults [32]. This highlights the importance of healthcare professionals’ need for knowledge, skills and appropriate methods in order to communicate with children of all ages and with different needs and abilities in order to enhance their participation in healthcare [33, 34]. In accordance with our results, the use of an e-health communication tool, Sisom, played a significant role in the work of supporting healthcare professionals for increasing their ability to allow the child to become an agent in her/his healthcare. Children aged 6–12 participated in our study, but even younger children have been shown to have the ability to use interactive communication tools in healthcare [15].

In accordance with the UNCRC (1989) and the Swedish Patient Act [25], there is an ambition in pediatric healthcare to be true to the principle of autonomy for children and involve them in decisions regarding their own healthcare and routinely ensuring that they are given a voice [28, 32, 35]. Even though children between 6 and 12 years are considered to be able to communicate their preferences, their voices are often unheard both in terms of communication with healthcare professionals and in terms of theoretical models created around communication roles in children’s healthcare. Parents are often seen as providers of information and intermediaries for communication for their children in the dialogue with healthcare professionals by both children [36, 37] and parents [37]. The focus in pediatric healthcare is thus still primarily on parents and healthcare professionals only share some information with the children instead of sharing decisions [30, 38]. However, children want to be included in the dialogue with healthcare professionals [36]. The goal of pediatric professionals is to guide parents and create opportunities for optimizing participation for their children in order to fulfill the ethical duties of adequate information provision and respect for children’s developing autonomy [39]. This can be a challenge as parents often want to protect their children from the emotional stress that can arise as a part of decision-making by protecting them from disturbing information about the illness and treatment to maintain the child’s hope and spirits [37, 39, 40]. This need for protecting their children was, however, not found in our study. In contrast, the parent’s ambition was for their children to achieve independence and they claimed that the children’s self-esteem increased when they were easily able to express themselves with Sisom. A possible explanation may be that the parents in our study became aware of how much information the children can give themselves by using Sisom and how much information the parents discovered they did not know about their child. The parents experienced emotional relief when the focus of the dialogue with healthcare professionals turned towards the child instead of keeping the parents responsible for knowing and making decisions about their child’s best interests.

An interesting finding from our study, in addition to increasing the child’s participation, was that the use of Sisom also helped the children to start to process their feelings and emotions in relation to their illness and treatment. When the child answered questions in Sisom, both awareness of the child’s inner thoughts and feelings was raised as well as their existence is acknowledged. Revealing feelings was sometimes difficult for the child and could cause both sadness and tiredness afterward, although the parents considered it important for their child’s processing of the situation, which is also confirmed by Baggot et al. [20]. Thus, putting feelings into words is important for reducing the risk of creating unspeakable suffering for the children [41].

## Conclusion

Orientating communication about healthcare towards the voice of the child with Sisom was experienced by the parents as a key prerequisite for increasing their children’s participation in healthcare. The children became more independent of their parents when they were given the opportunity to express themselves in an easy and child-friendly way and were thus given the opportunity to grow as human beings. This resulted in relief among the parents as their responsibility for being an advocate for their child was reduced. The parents also highlighted that the use of Sisom could initiate a process enabling their child to cope with experiences of illness and treatment. The conclusion is that the use of Sisom reveals the children’s perspectives of themselves and thus facilitate provision of child-centered care.

## Data Availability

Not applicable. The data will not be shared as ethics approval for the study requires that the transcribed interviews are kept in locked files, accessible only to the researchers.

## Abbreviations

Sisom: (Norwegian acronym “Si det som det er” or “Tell it how it is”)
UNCRC: United Nations Convention on the Rights of the Child
